# Time trends in prescribing of type 2 diabetes drugs, glycaemic response and risk factors: A retrospective analysis of primary care data, 2010–2017

**DOI:** 10.1111/dom.13687

**Published:** 2019-04-04

**Authors:** John M. Dennis, William E. Henley, Andrew P. McGovern, Andrew J. Farmer, Naveed Sattar, Rury R. Holman, Ewan R. Pearson, Andrew T. Hattersley, Beverley M. Shields, Angus G. Jones, on behalf of the MASTERMIND consortium

**Affiliations:** ^1^ Health Statistics Group Institute of Health Research, University of Exeter Medical School Exeter UK; ^2^ Institute of Biomedical and Clinical Science Royal Devon and Exeter Hospital Exeter UK; ^3^ Nuffield Department of Primary Care Health Sciences University of Oxford Oxford UK; ^4^ Institute of Cardiovascular and Medical Sciences University of Glasgow Glasgow UK; ^5^ Diabetes Trials Unit, Oxford Centre for Diabetes, Endocrinology and Metabolism University of Oxford Oxford UK; ^6^ Division of Molecular and Clinical Medicine, Ninewells Hospital and Medical School University of Dundee Dundee UK

**Keywords:** type 2 diabetes, SGLT2 inhibitor, primary care, weight control, glycaemic control, hypoglycaemia

## Abstract

**Aim:**

To describe population‐level time trends in prescribing patterns of type 2 diabetes therapy, and in short‐term clinical outcomes (glycated haemoglobin [HbA1c], weight, blood pressure, hypoglycaemia and treatment discontinuation) after initiating new therapy.

**Materials and methods:**

We studied 81 532 people with type 2 diabetes initiating a first‐ to fourth‐line drug in primary care between 2010 and 2017 inclusive in United Kingdom electronic health records (Clinical Practice Research Datalink). Trends in new prescriptions and subsequent 6‐ and 12‐month adjusted changes in glycaemic response (reduction in HbA1c), weight, blood pressure and rates of hypoglycaemia and treatment discontinuation were examined.

**Results:**

Use of dipeptidyl peptidase‐4 inhibitors as second‐line therapy near doubled (41% of new prescriptions in 2017 vs. 22% in 2010), replacing sulphonylureas as the most common second‐line drug (29% in 2017 vs. 53% in 2010). Sodium‐glucose co‐transporter‐2 inhibitors, introduced in 2013, comprised 17% of new first‐ to fourth‐line prescriptions by 2017. First‐line use of metformin remained stable (91% of new prescriptions in 2017 vs. 91% in 2010). Over the study period there was little change in average glycaemic response and in the proportion of people discontinuing treatment. There was a modest reduction in weight after initiating second‐ and third‐line therapy (improvement in weight change 2017 vs. 2010 for second‐line therapy: −1.5 kg, 95% confidence interval [CI] −1.9, −1.1; *P* < 0.001), and a slight reduction in systolic blood pressure after initiating first‐, second‐ and third‐line therapy (improvement in systolic blood pressure change 2017 vs. 2010 range: −1.7 to −2.1 mmHg; all *P* < 0.001). Hypoglycaemia rates decreased over time with second‐line therapy (incidence rate ratio 0.94 per year, 95% CI 0.88, 1.00; *P* = 0.04), mirroring the decline in use of sulphonylureas.

**Conclusions:**

Recent changes in prescribing of therapy for people with type 2 diabetes have not led to a change in glycaemic response and have resulted in modest improvements in other population‐level short‐term clinical outcomes.

## 1 INTRODUCTION

Prescribing of glucose‐lowering therapies for patients with type 2 diabetes has changed markedly in recent years. International guidelines have been updated to include a much greater choice of agents when additional therapies after metformin are required to achieve glycaemic control.^1^, ^2^, ^3^, ^4^ Newer drug classes including dipeptidyl peptidase‐4 (DPP‐4) inhibitors, sodium‐glucose co‐transporter‐2 (SGLT2) inhibitors and glucagon‐like peptide‐1 (GLP‐1) receptor agonists are now established alongside the longstanding options sulphonylureas, thiazolidinediones and insulin. Choice between these agents is left largely to the clinician and patient. Recent studies show that there have been marked changes in which agents are initiated after metformin, with declining use of sulphonylureas and increasing and earlier use of DPP‐4 inhibitors and SGLT2 inhibitors in both the United States, Europe and the United Kingdom.^5^, ^6^, ^7^, ^8^

Although studies have suggested that the glucose‐lowering effectiveness of agents typically added to metformin may be comparable,^1^, ^9^, ^10^ there are well established differences between the different drug classes in weight change and side effects. GLP‐1 receptor agonists and SGLT2 inhibitors are associated with weight loss, whereas DPP‐4 inhibitors are weight‐neutral and sulphonylureas can promote weight gain.^9^, ^10^ Hypoglycaemia risk is greater with sulphonylureas and insulin relative to other agents.^9^ Despite these known differences in non‐glycaemic effects between agents, evidence of the impact of recent changes in prescribing on population‐level patient outcomes is limited.^5^, ^7^, ^11^, ^12^ In the present study we aimed to describe changes in prescribing of glucose‐lowering drugs for patients initiating first‐ to fourth‐line therapy between 2010 and 2017 in the United Kingdom, a setting where prescribing does not reflect the ability of patients to pay. We further examined population‐level time trends in the short‐term clinical outcomes of glycaemic response, weight change, blood pressure change, hypoglycaemia and treatment discontinuation.

## 2 MATERIALS AND METHODS

### 2.1 Data source and data extraction

We conducted a population‐based analysis of anonymized primary care data from the UK Clinical Practice Research Database (CPRD). CPRD is a population‐representative database including demographic, clinical and prescription primary care records of patients.^13^ Although CPRD includes full prescription records, no data on drug dispensation are available. CPRD has been extensively used to study drug prescribing and patient outcomes in type 2 diabetes.^14^ We analysed data from the January 2018 release of CPRD, including all practices that were still contributing to CPRD in 2017, to ensure that changes in prescribing did not reflect changes in the practices captured in CPRD over the study period. We classified glucose‐lowering drugs into drug classes according the British National Formulary sections 6.1.1 and 6.1.2.^15^ Drugs were categorized as metformin, sulphonylureas, thiazolidinediones, DPP‐4 inhibitors, GLP‐1 receptor agonists, SGLT2 inhibitors, insulin or other (meglitinides and α‐glucosidase inhibitors, which are prescribed very rarely in the United Kingdom). Scientific approval was granted by the CPRD Independent Scientific Advisory Committee (ISAC 13_177RA4R).

### 2.2 Study population

We extracted the clinical and prescription records of all patients with type 2 diabetes who started at least one glucose‐lowering drug for the first time ever between 1 January 2010 and 31 December 2017 and met CPRD quality assurance criteria. Inclusion criteria and data ascertainment were the same as those included in our previously reported CPRD cohort profile.^16^ Type 2 diabetes was defined largely on the basis of prescriptions for non‐insulin diabetes therapies rather than diagnostic medical codes, to minimize coding errors.^17^ We excluded patients with diagnostic codes for other forms of diabetes or polycystic ovary syndrome, which can be treated with metformin. To remove patients with type 1 diabetes, whose disease may have been miscoded as type 2, we excluded patients with an age at diagnosis of <35 years or who were on insulin treatment within 12 months of diagnosis. Consequently, patients with type 2 diabetes whose first‐line therapy was insulin were not included. We defined date of diabetes diagnosis as the earliest of: first prescription for a non‐insulin diabetes therapy; first glycated haemoglobin (HbA1c) result ≥48 mmol/mol (6.5%); or first diabetes diagnostic code.

### 2.3 Study design

The study exposure was a new first‐ to fourth‐line drug prescription record for a patient within the study period. New drug prescriptions (and their corresponding start dates) were defined as the first‐ever prescription of a drug in a class for a patient. First‐, second‐, third‐ or fourth‐line prescription categories were defined based on the order of new drug prescriptions for individual patients. Every time a patient started a new drug class we assigned this to the next line of therapy, regardless of whether their concomitant therapy changed at a similar time point.

The primary unit of analysis was line of therapy. This meant individual patients who started more than one new therapy over the study period contributed to the analysis more than once with different lines of therapy (Flowchart S1).

### 2.4 Study outcomes

For each line of therapy, we evaluated annual time trends in the drug classes initiated, and time trends in changes in HbA1c, weight, systolic and diastolic blood pressure, hypoglycaemia rates and treatment discontinuation after therapy start. To evaluate all outcomes we used a “new‐user” design, which mitigated immortal time bias.^18^ Patients were followed up from their drug start date until there was any change in diabetes therapy or the end of the study period specific to each outcome. A change in therapy could be the addition of a new glucose‐lowering drug or the stopping of the drug of interest or any concomitant glucose‐lowering drug. Patients were considered to have stopped a drug if there was a subsequent gap in prescribing of that drug for at least 6 months.^16^

We defined glycaemic response (the change in HbA1c), weight change and blood pressure change as the absolute change from baseline to 6 months (6‐month measure minus baseline measure). For glycaemic response, baseline HbA1c was defined as the closest HbA1c to the drug start date in the 3 months prior to the drug start date. HbA1c at 6 months was defined as the closest HbA1c value to 6 months after the drug start date (±3 months). Glycaemic response was only valid if there were no changes in glucose‐lowering therapy between 2 months prior to the baseline HbA1c and the date of the 6‐month HbA1c. The same approach was used for weight change and blood pressure change.

We defined hypoglycaemia as the first Read code for hypoglycaemia up to 2 years after starting a line of therapy, using a previously published Read code list for hypoglycaemia.^19^ Because of the low number of hypoglycaemia events captured in primary care we grouped data into biannual categories representing four distinct periods (2010‐2011, 2012‐2013, 2014‐2015, 2016‐2017).

We examined treatment discontinuation by estimating the proportion of patients who stopped a therapy within 3 months, 6 months and 1 year. Six months' follow‐up after discontinuation was required to determine no new prescriptions were issued.

### 2.5 Statistical analysis

We examined annual time trends for each clinical outcome and each line of therapy in separate analysis. We described trends in baseline clinical characteristics as mean (SD) per calendar year. All outcomes analyses were standardized to the mean baseline values of relevant measures for patients starting that line of therapy in 2017.

To evaluate changes in relative prescribing for each line of therapy we calculated the proportion of new prescriptions for each drug class in each calendar year as the:

$$\frac{\text{total number of}\;\mathrm{new}\;\text{prescriptions of the drug}}{\text{total number of}\;\mathrm{new}\;\text{prescriptions}}$$

When describing first‐line therapy, all drugs except metformin and sulphonylureas were pooled. Within‐drug‐class trends for DPP‐4 inhibitors, GLP‐1 receptor agonists, SGLT2 inhibitors and sulphonylureas over 2014‐2017 were estimated using the same approach.

We evaluated non‐linear time trends in glycaemic response, weight change and blood pressure change for each calendar year using linear regression, with calendar year as a categorical covariate and adjustment for baseline HbA1c, age at therapy, duration of diabetes, and the baseline measure of the outcome for non‐glycaemic outcomes. We used complete case analysis including patients only if they had both a valid baseline measure and a valid 6‐month measure. To assess the potential influence of missing data, we compared the characteristics of the patients with missing data with those included in the analysis. Multiple imputation was not conducted as it is only valid under the missing‐at‐random assumption (meaning the differences between the observed and missing data could be explained by other recorded measures), and we felt missing outcome data were likely to depend on their actual value (missing not at random). Hypoglycaemia biannual time trends were estimated as rates per 1000 person‐years using Poisson regression, adjusted for age, duration and baseline HbA1c level.

Summary measures for each outcome (including baseline HbA1c) were calculated as follows: (a) the 2017 vs. the 2010 marginal contrast from the multivariable linear regression models described above^20^; (b) the linear time trend, as the β coefficient from a multivariable linear regression, treating calendar year as a continuous rather than categorical covariate.

To evaluate changes in treatment discontinuation we calculated the proportion of new prescriptions that were stopped within 3 months, 6 months and 1 year for each line of therapy for each calendar year as:

$$\frac{\text{total number of}\;\mathrm{new}\;\text{prescriptions stopped within time period}\;}{\text{total number of}\;\mathrm{new}\;\text{prescriptions}}$$

All data extraction and analysis was conducted in STATA v14.0.

### 2.6 Sensitivity analysis

We repeated all outcomes analysis using change in each measure from baseline to 12 months as the outcome in a distinct cohort of patients with 12‐month measures of each outcome (closest ±3 months as for the definition of 6‐month change). Participants commencing therapy in 2017 were not included in this analysis as 12 months of patient follow‐up had not accrued. We also evaluated the sensitivity of results to our definition of line of therapy by repeating all second‐line analyses in a subset of patients who were initiated on metformin first‐line and then added a different therapy to metformin (rather than stopping metformin). To assess whether changes in outcomes over time were likely to be attributable to changes in the drugs prescribed, we compared time trends in weight change and hypoglycaemia using the same models described above, with drug as an additional covariate.

## 3 RESULTS

A total of 123 990 new first‐ to fourth‐line prescriptions for 81 532 individual patients were eligible for inclusion, of which 40% (50 215) were for a first‐line prescription, 26% (32 071) were second‐line, 20% (25 024) were third‐line and 13% (16 680) were fourth‐line (Flowchart S1). The baseline clinical characteristics of patients starting each line of therapy in 2017 are shown in Table S1. The mean baseline HbA1c at which second‐ to fourth‐line therapy was initiated increased over the study period; mean baseline weight increased first‐line, but there was little difference for other lines of therapy. The proportion of patients with valid measures for inclusion in the analysis of each outcome is shown in the Flowchart S1.

### 3.1 Changing prescribing of glucose‐lowering therapy

**Figure 1:**
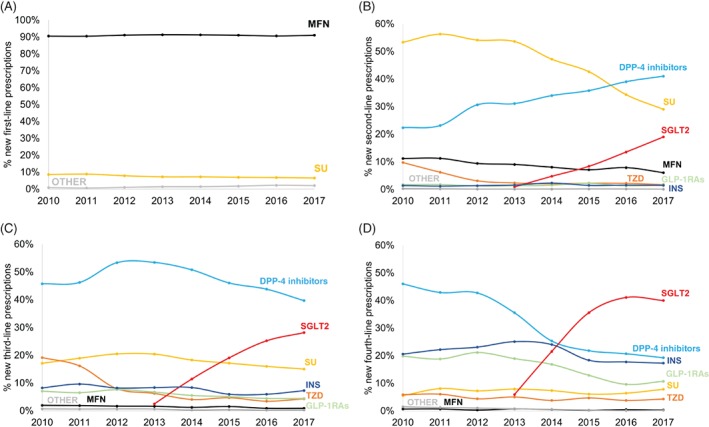
Time trends in new drug prescriptions for A, first‐line, B, second‐line, C, third‐line and D, fourth‐line therapy. The prescriptions for each drug class each year are given as a percentage of total new drug prescriptions for that year. DPP‐4, dipeptidyl peptidase‐4; GLP‐1RAs, glucagon‐like peptide‐1 receptor agonists; INS, insulin; MFN, metformin; SGLT2, sodium‐glucose co‐transporter‐2 inhibitors; SU, sulphonylureas; TZD, thiazolidinediones

We found marked changes in relative prescribing of second‐ to fourth‐line therapy (Figure 1). DPP‐4 inhibitors were, by 2017, the most commonly initiated second‐line therapy (41% of new second‐line therapies in 2017 vs. 22% of new second‐line therapies in 2010), whilst second‐line prescribing of sulphonylureas decreased (29% in 2017 vs. 53% in 2010). SGLT2 inhibitors were the most common fourth‐line therapy in 2017 (40% of prescriptions) and their use second‐line (19% of new 2017 prescriptions) and third‐line (28% of new 2017 prescriptions) increased rapidly following their introduction in 2013. Fourth‐line prescribing of injectable therapy decreased (GLP‐1 receptor agonists: 11% in 2017 vs. 20% in 2010; insulin: 17% in 2017 vs. 21% in 2010), and remained low second‐ and third‐line. First‐line use of metformin remained stable (91% in 2017 vs. 91% in 2010).

Evaluating new first‐ to fourth‐line drug initiations as a whole (Figure S1), we found SGLT2 inhibitors (17% of total new prescriptions in 2017) were more commonly initiated in 2017 than sulphonylureas (14% in 2017). New prescribing of insulin (5% in 2017 vs. 5% in 2010) and GLP‐1 receptor agonists (range 4%‐3%) remained constant over the study period.

### 3.2 Changes in within‐class prescribing

In addition to changes in class of agent there have been marked recent changes in prescription of individual agents within a class. From 2014 to 2017 for DPP‐4 inhibitors, there was decreasing use of sitagliptin (37% in 2017 vs. 56% in 2014), but increasing use of alogliptin (25% in 2017 vs. 1% in 2014) and linagliptin (31% in 2017 vs. 25% in 2014; Figure S2A). For GLP‐1 receptor agonists, use of once‐weekly dulaglutide increased to 51% of the class total following its introduction in 2015 (Figure S2B). For SGLT2 inhibitors, there was increasing use of empagliflozin (46% in 2017 vs. 8% in 2015), but decreasing use of dapagliflozin (41% in 2017 vs. 92% in 2014; Figure S2C). Gliclazide use has remained stable (91% of all sulphonylureas in 2017 vs. 89% in 2010; Figure S2D).

### 3.3 Reduction in HbA1c

**Figure 2:**
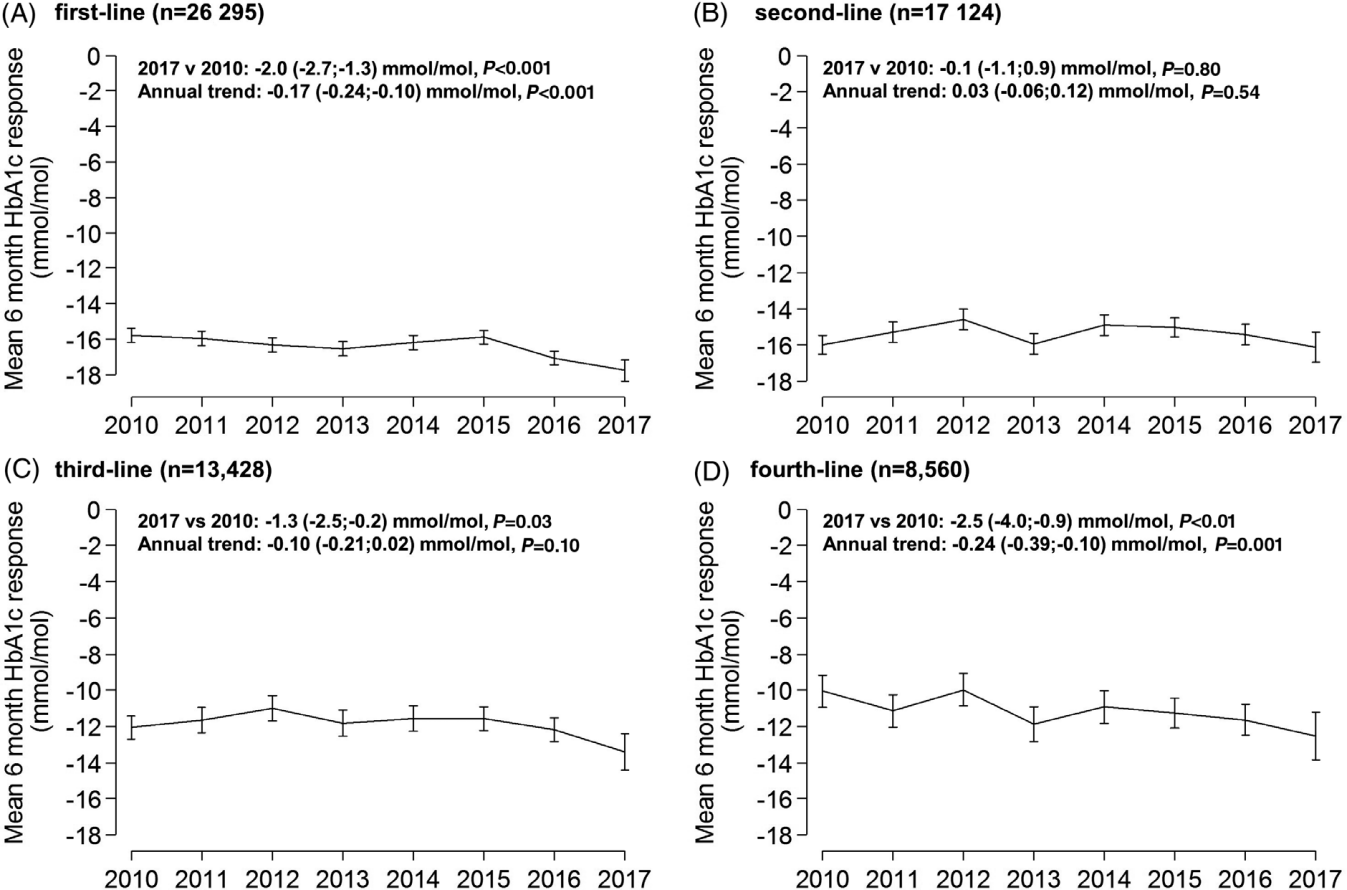
Mean glycated haemoglobin (HbA1c) response at 6 months, 2010‐2017, for A, first‐line, B, second‐line, C, third‐line and D, fourth‐line therapy. Error bars are 95% confidence intervals. Data are standardized to the average baseline HbA1c, age at diagnosis and duration of diabetes, specific to each drug line in 2017

Average reductions in HbA1c at 6 months were relatively constant over 2010 to 2017 across all lines of therapy (Figure 2). There was no evidence of a change in glycaemic response for second‐line therapy (2017 vs. 2010 change −0.1 mmol/mol [0.0%]; *P* = 0.80). For first‐, third‐ and fourth‐line therapy there was evidence of a statistically significant trend towards improved glycaemic response, although this translated to a modest absolute improvement in reduction in HbA1c (2017 vs. 2010 change range 1.3‐2.5 mmol/mol [0.2%‐0.3%]; all *P* < 0.05).

### 3.4 Weight change

**Figure 3:**
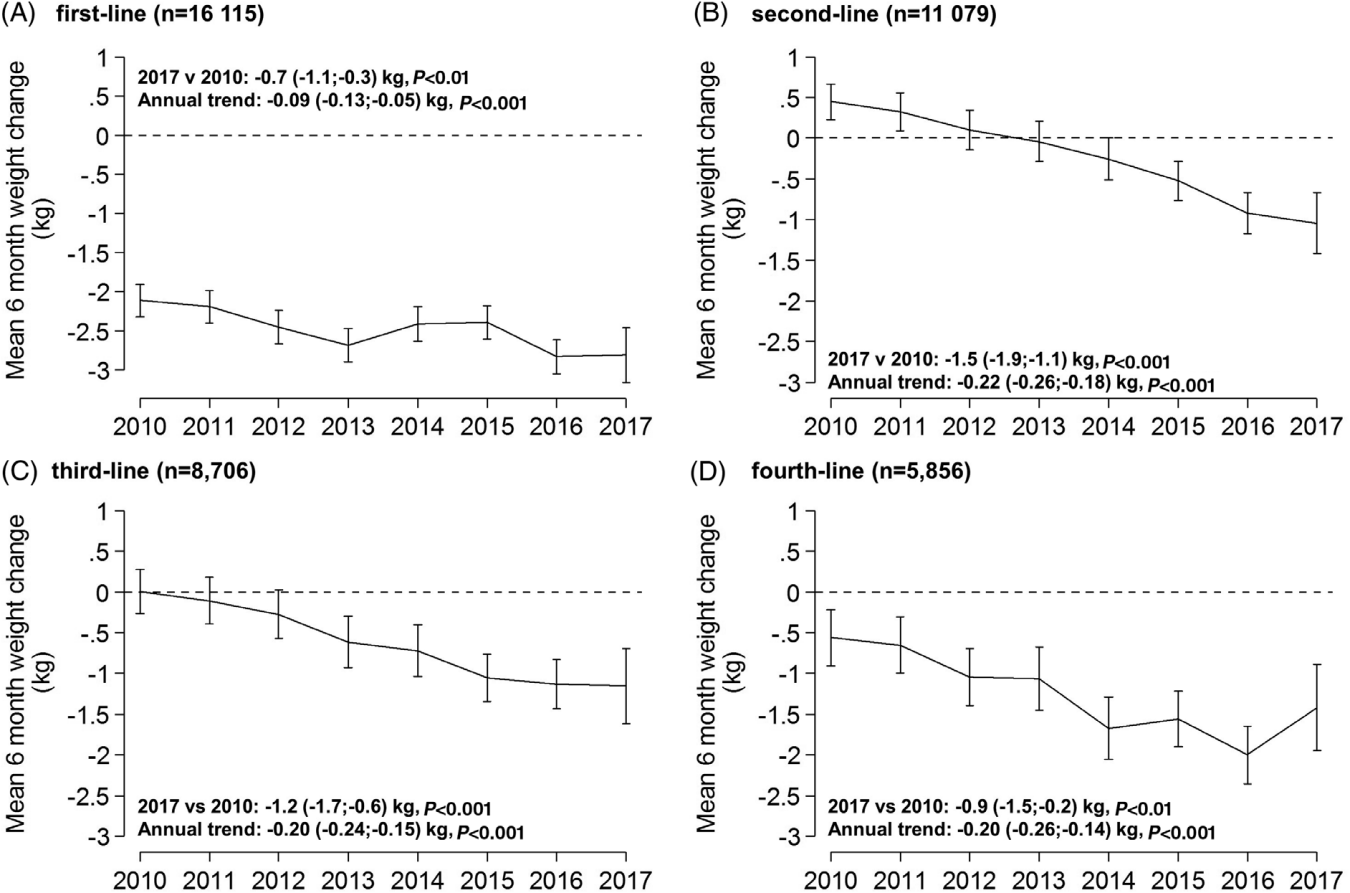
Mean change in weight at 6 months, 2010 to 2017 for A, first‐line, B, second‐line, C, third‐line and D, fourth‐line therapy. Error bars are 95% confidence intervals. Data are standardized to the average baseline weight, baseline HbA1c, age at diagnosis and duration of diabetes, specific to each drug line in 2017

Although there was a trend towards greater weight loss at 6 months for all lines of therapy, this was most marked with second‐ and third‐line therapy (2017 vs. 2010, second‐line −1.5 kg and third‐line −1.2 kg; both *P* < 0.001, overall time trends for improvement in weight change *P* < 0.001 for all lines of therapy [Figure 3]). Patients starting second‐line therapy on average lost rather than gained weight when comparing 2017 with 2010.

### 3.5 Blood pressure

We found a trend towards a modest improvement in systolic blood pressure at 6 months for all lines of therapy (2017 vs. 2010 range − 1.7 to −2.1 mmHg, all *P* < 0.001 [Figure S3A]). There was no change in diastolic blood pressure (Figure S3B).

### 3.6 Hypoglycaemia

**Figure 4:**
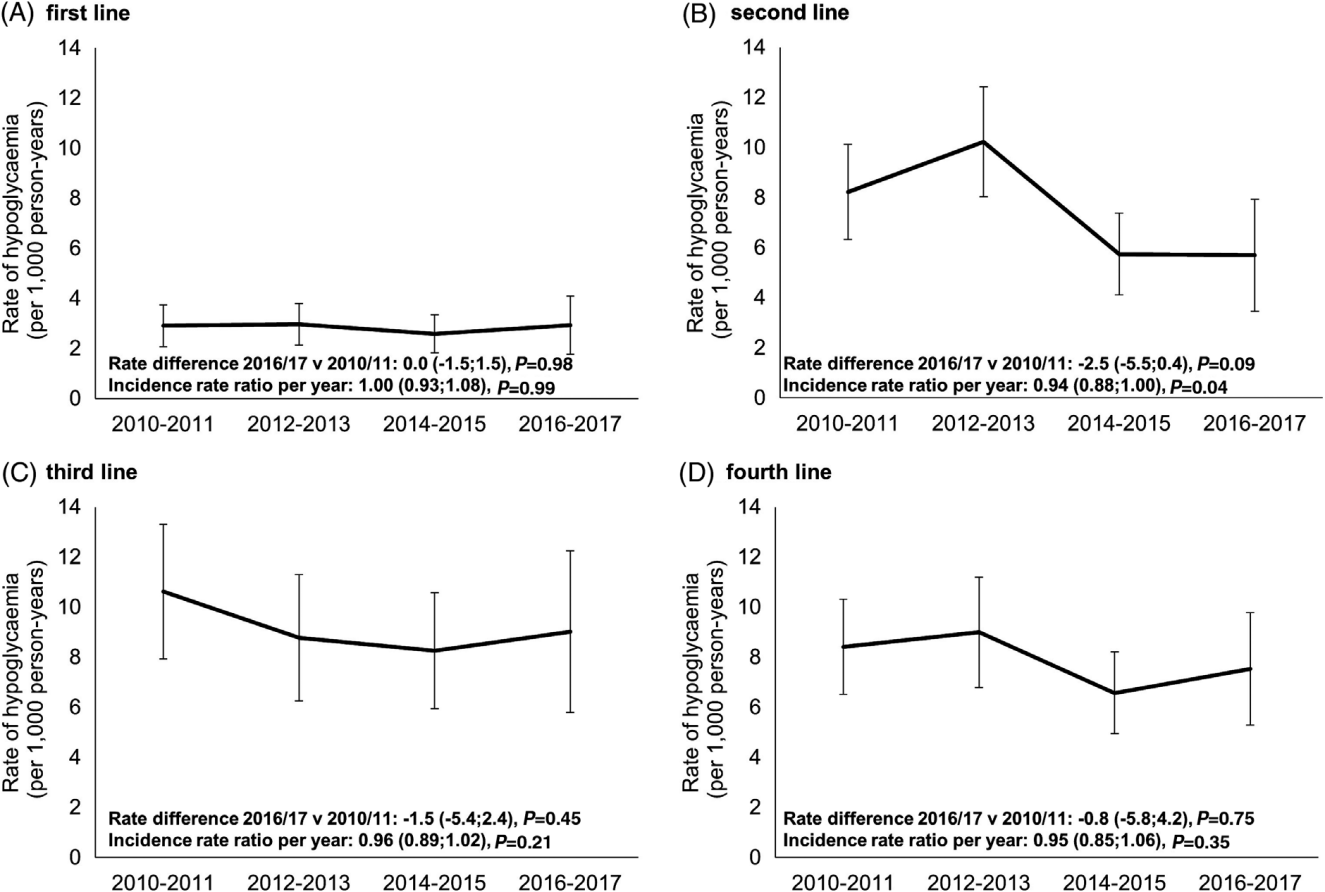
Hypoglycaemia rates per 1000 person‐years by 2‐year period for A, first‐line, B, second‐line, C, third‐line and D, fourth‐line therapy**.** Rates represent the occurrence of hypoglycaemia over the first 2 years after starting a line of therapy

We observed a decrease in hypoglycaemia rates for patients starting second‐line therapy (2017 rate 5.7 per 1000 person‐years, 95% confidence interval [CI] 3.5, 7.9; 2010 rate 8.2 per 1000 person‐years, 95% CI 6.3, 10.1 [Figure 4 and Table S2]).

### 3.7 Treatment discontinuation

Treatment discontinuation at 3 months, 6 months and 1 year after initiating therapy was stable over the period 2010 to 2017 (Table S3). The proportion of patients discontinuing within 3 months in 2017 compared to 2010 was as follows: first‐line 4% vs 3%; second‐line 7% vs 9%; third‐line 12% vs 9%; fourth‐line 10% vs 9%.

### 3.8 Sensitivity analysis

The baseline characteristics of the patients who were excluded as they did not have valid clinical measures were similar to those included in the analysis (Table S4). Time trends for outcomes at 12 months were similar to those at 6 months for glycaemic response (Figure S4), weight change (Figure S5) and blood pressure (Figure S6). Second‐line prescribing trends and patient outcomes in the subset of patients adding a second‐line drug to continued first‐line metformin therapy (73% of patients included in the primary analysis) were near identical to those in the primary analysis (Figure S7). Weight change trends were reduced when models were adjusted for drug therapy as a covariate (Table S5A) and, after adjustment for drug, there was no evidence of a difference in risk of hypoglycaemia over time (Table S5B).

## 4 DISCUSSION

The present study describes, for the first time, recent population‐level time trends in the short‐term clinical outcomes of patients initiating glucose‐lowering therapy over 2010 to 2017, a period in which there were substantial changes in type 2 diabetes prescribing patterns. There were modest population‐level improvements in weight change and rates of hypoglycaemia for patients starting additional therapy after metformin, but little change in glycaemic response, blood pressure change or treatment discontinuation. Information on these important clinical outcomes provide timely context to the worldwide trend towards prescribing of newer more costly glucose‐lowering agents. We also provide updated information on UK prescribing trends: (a) increased and earlier initiation of DPP‐4 inhibitors; (b) reduced initiation of sulphonylureas as second‐line therapy; (c) a rapid increase in initiation of SGLT2 inhibitors; and (d) decreased initiation of injectable therapy (GLP‐1 receptor agonists and insulin).

Whilst our retrospective analysis precludes causal inference and can only show temporal correlation, the time trends in patient outcomes reflect known effects of the different drug classes on clinical outcomes. As might be expected from previous comparative analysis,^9^, ^10^ there was an improvement in weight change and reduction in rates of hypoglycaemia where there was a rapid increase in the use of SGLT2 inhibitors and DPP‐4 inhibitors in place of sulphonylureas. These changes were attenuated by statistical adjustment for drug, supporting the suggestion that the population‐level improvements relate to changes in prescribing. Although recent meta‐analyses have found little difference in glycaemic response when comparing therapies added to metformin,^1^, ^9^ some studies have reported increased response with sulphonylureas compared with other agents,^21^, ^22^, ^23^ or lower response with DPP‐4 inhibitors,^10^ and so it is reassuring that we found second‐line glycaemic response was stable despite the changes in prescribing. Newer agents, in particular SGLT2 inhibitors, have been associated with modestly lower blood pressure.^24^, ^25^, ^26^, ^27^ However, whilst there were small improvements over time in blood pressure change with second‐ to fourth‐line therapy, there were also improvements first‐line where prescribing was unaltered. This suggests that improvements do not solely reflect changes in prescribing of glucose‐lowering medication.

The trends in new prescribing observed in the present study are consistent with previous United Kingdom primary care data,^7^ including a recent analysis which also documented extensive geographical variation in prescribing.^6^ Comparison with data from the United States suggests newer therapies have been adopted more quickly in the United Kingdom; in the United States, sulphonylureas remain the most common second‐line therapy.^5^ However, time trends in new prescribing are similar; in the United States there has been decreasing second‐line use of sulphonylureas (46% of new second‐line prescriptions in 2016 vs. 55% in 2010) and increasing use of DPP‐4 inhibitors (20% in 2016 vs. 14% in 2010). The higher cost of newer agents may explain their relatively slower uptake in the United States.^5^

There have been few recent studies examining time trends of patient outcomes. A recent analysis of 1.7 million United States Medicare patients found no overall population‐level change in glycaemic control or rates of hypoglycaemia over 2006 to 2013 but, unlike the present study, did not examine the outcomes of patients initiating new therapy.^12^ Declining overall rates of hypoglycaemia requiring hospitalization were observed in UK patients aged >65 years, but not in those aged <65 years, from 2009 to 2013, in the context of declining use of sulphonylureas in this older age group.^28^ The changes observed in these studies examining the overall population of patients with type 2 diabetes will lag considerably behind those observed in the present analysis of new therapy initiation, as, once initiated, a glucose‐lowering therapy may be continued for decades.

Strengths of the present study include our approach examining new prescribing, which allowed interrogation of time trends whilst accounting for the increasing prevalence of type 2 diabetes, which in the United Kingdom is attributable more recently to declining mortality rather than increasing incidence,^29^, ^30^ and means prescribing of glucose‐lowering therapy is increasing in absolute terms.^6^, ^31^ Our definition of type 2 diabetes should minimize misclassification.^16^ The present study provides a near‐complete picture of United Kingdom prescribing because, in the United Kingdom, type 2 diabetes is largely managed in primary care. Even new therapy initiated on the advice of a specialist will usually be prescribed by the patients' primary care physician. A limitation of the present study is the weakness in the way hypoglycaemia is recorded. It is likely that many episodes of hypoglycaemia will be missing from a patients' primary care record, as mild hypoglycaemia or more severe hypoglycaemia requiring attendance in secondary care are poorly recorded; however, previous studies have provided useful insight into hypoglycaemia using similar definitions in the same dataset.^32^ Although the missing records mean the absolute rates of hypoglycaemia in the present study will be an underestimate, the specificity of our key finding, a relative decrease in hypoglycaemia rates with second‐line therapy, where use of sulphonylureas has markedly declined, is reassuring. Whilst the present study provides timely information on population‐level trends, further observational studies, building on recent work, will be needed to establish the real‐world comparative effectiveness of individual drug classes at different lines of therapy.^10^, ^33^

Our results show that prescribing of glucose‐lowering therapy in Type 2 diabetes is rapidly changing towards newer, more expensive agents. Changes in prescribing appear to have pre‐empted rather than reflected changes in clinical guidelines.^1^ In particular, second‐line prescribing of DPP‐4 inhibitors increased rapidly long before treatment guidelines were updated to position them along sulphonylureas and pioglitazone as second‐line options.^1^ The positive trends in weight change, hypoglycaemia and blood pressure are likely to have improved the quality of life for patients, and a reduction in hypoglycaemia is also likely to have a cost benefit.^34^ However, given the much higher cost of newer drug options, the modest improvements we observed in patient outcomes suggests further studies are needed to evaluate cost‐effectiveness of the newer glucose‐lowering agents. Recent evidence suggests there may be potential for a more stratified approach to prescribing of type 2 diabetes therapy, meaning prescribing decisions can be better informed through identification of patients or subgroups who differ in their likely glycaemic response or risk of side effects with individual agents.^2^, ^35^, ^36^

We did not evaluate microvascular or macrovascular outcomes in the present study, but a cardiovascular benefit in individuals with established cardiovascular disease, has recently been demonstrated in placebo‐controlled trials with SGLT2 inhibitors and GLP‐1 receptor agonists.^24^, ^37^, ^38^ A recent meta‐analysis of randomized trials suggested that, in contrast to SGLT2‐inhibitors and GLP‐1 receptor agonists, there is no short‐term mortality benefit with DPP‐4 inhibitors.^39^ Given the recent changes in treatment guidelines to consider cardiovascular risk when choosing therapy,^4^ and the fact all three classes have now been prescribed in significant numbers for some years, an evaluation of recent time trends in microvascular and macrovascular complications would be of considerable interest.

In conclusion, the trend towards prescribing of newer, more expensive, glucose‐lowering medication in the United Kingdom has coincided, for patients initiating new therapy, with a likely reduction in hypoglycaemia rates and a modest improvement in weight and blood pressure, but little change in glycaemic response or treatment discontinuation. These results demonstrate the potential population‐level impact of the rapid changes that are occurring in prescribing of glucose‐lowering therapy worldwide.

## CONFLICT OF INTEREST

W.E.H. has received a grant from IQVIA. A.P.M. has received grants from Eli Lilly and Pfizer. E.R.P. has received personal fees from Eli Lilly, MSD and Novo Nordisk. N.A.S. has received personal fees from Amgen, Astra Zeneca, Boehringer Ingelheim, Eli Lilly, Janssen, Sanofi, Novo Nordisk, and a grant from Boehringer Ingelheim. R.H.H. has received personal fees from Bayer, Boehringer Ingelheim, Novartis, Amgen, Elcelyx, GSK, Jannsen, Servier and Takeda. Representatives from GSK, Takeda, Janssen, Quintiles, AstraZeneca and Sanofi attend meetings as part of the industry group involved with the MASTERMIND consortium. No industry representatives were involved in the writing of the manuscript or analysis of data. For all authors these fees/grant were outside the submitted work; no other relationships or activities that could appear to have influenced the submitted work are declared.

## AUTHOR CONTRIBUTIONS

J.M.D., A.T.H., B.M.S. and A.G.J. designed the study. J.M.D. and B.M.S. analysed the data. J.M.D., B.M.S. and A.G.J. drafted the article. A.T.H., A.P.M., A.J.F., E.R.P., N.S., W.E.H. and R.H.H. provided support for the analysis and interpretation of results, and critically revised the article. All authors had full access to all of the data and take responsibility for the integrity of the data and the accuracy of the data analysis. B.M.S. and A.G.J. are the guarantors.

## Supporting information

Appendix
