# Supplementary material for: Time trends in prescribing of type 2 diabetes drugs, glycaemic response and risk factors: A retrospective analysis of primary care data, 2010–2017
Source: Diabetes Obes Metab. 2019 Apr 4;21(7):1576–84. doi: 10.1111/dom.13687 (PMC6618851; doi:10.1111/dom.13687)

**Supplementary Material for:**

**Time trends in prescribing of type 2 diabetes drugs, glycemic control response and risk factors: a retrospective analysis of primary care data, 2010-2017**

**Authors:** John M Dennis, William E Henley, Andrew P McGovern, Andrew J Farmer, Naveed Sattar, Rury R Holman, Ewan R Pearson, Andrew T Hattersley, Beverley M Shields, Angus G Jones on behalf of the MASTERMIND consortium

Supplementary Flowchart: Overview of patient flowchart, study design, and patients included in each analysis


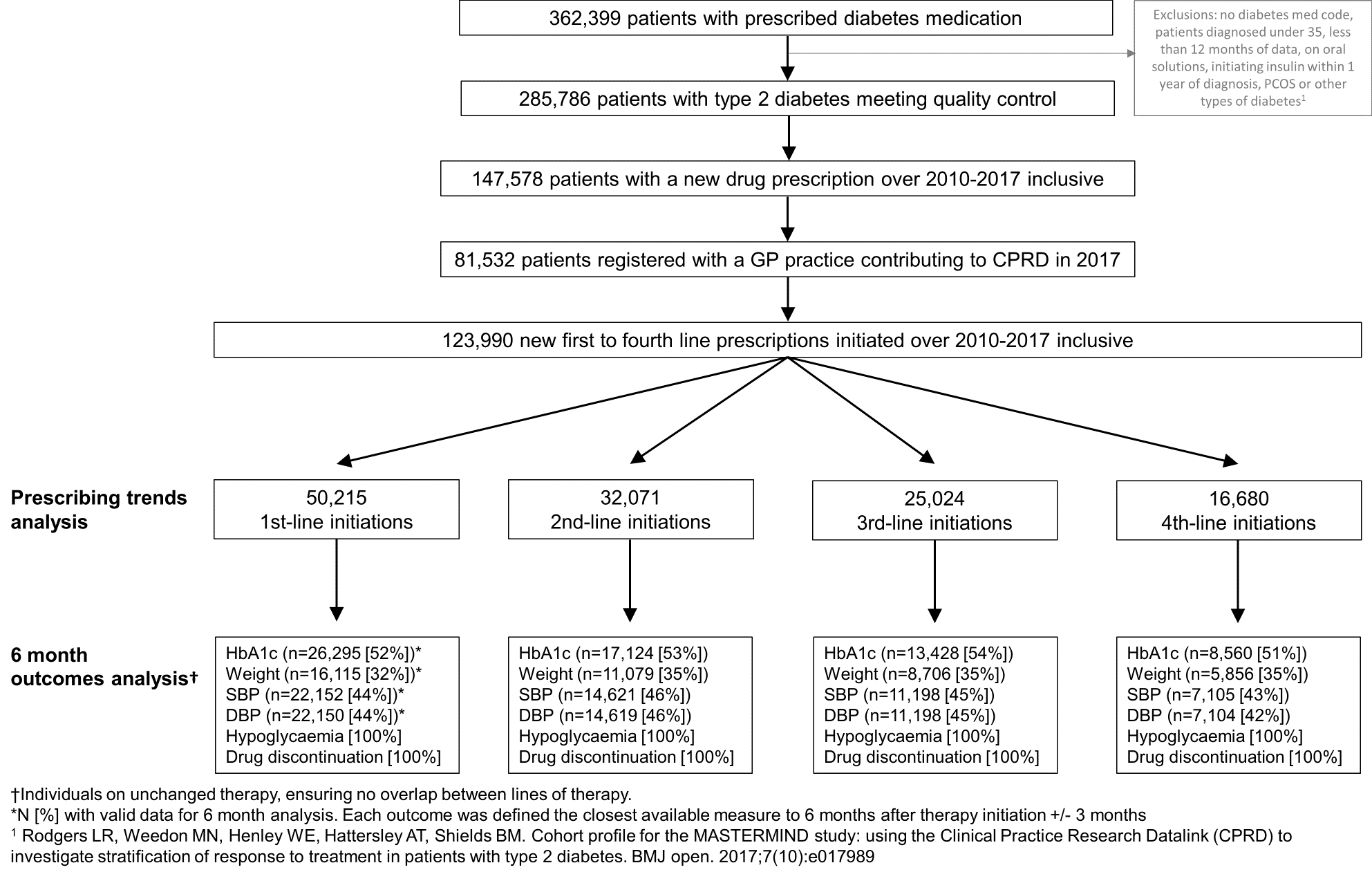


**Supplementary table 1: Average baseline clinical characteristics by calendar year and therapy (2010-2017)**

**a) first-line**

|  | 2010 | 2011 | 2012 | 2013 | 2014 | 2015 | 2016 | 2017 |
| --- | --- | --- | --- | --- | --- | --- | --- | --- |
| N | 6682 | 6252 | 6170 | 6341 | 5993 | 6719 | 6709 | 5349 |
| Baseline HbA1c (mmol/mol) | 71 (22) | 72 (22) | 73 (22) | 72 (22) | 71 (22) | 72 (23) | 72 (22) | 71 (22) |
| Age at therapy (years) | 62 (12) | 62 (12) | 62 (12) | 62 (12) | 62 (12) | 62 (12) | 62 (12) | 62 (12) |
| Duration of diabetes (years) | 2 (3) | 2 (3) | 2 (3) | 2 (3) | 2 (3) | 2 (3) | 2 (3) | 2 (3) |
| Sex (% Male) | 59% | 59% | 59% | 58% | 58% | 58% | 58% | 59% |
| Ethnicity (% White/missing) | 94% | 93% | 94% | 93% | 92% | 93% | 92% | 92% |
| BMI (kg/m²) | 33 (7) | 33 (7) | 33 (7) | 33 (7) | 33 (7) | 33 (7) | 33 (7) | 33 (7) |
| Weight (kg) | 93 (21) | 93 (21) | 94 (21) | 94 (22) | 94 (22) | 95 (22) | 95 (22) | 96 (22) |
| eGFR (mL/min/1.73 m²) | 80 (19) | 81 (19) | 82 (19) | 83 (19) | 83 (19) | 83 (19) | 83 (19) | 83 (18) |
| Systolic blood pressure (mmHG) | 137 (16) | 136 (16) | 136 (16) | 136 (16) | 135 (15) | 136 (16) | 136 (16) | 135 (16) |
| Diastolic blood pressure (mmHG) | 80 (10) | 80 (10) | 80 (10) | 79 (10) | 79 (10) | 79 (10) | 79 (10) | 80 (10) |
| HDL (mmol/L) | 2.8 (1) | 2.8 (1.1) | 2.9 (1) | 2.8 (1.1) | 2.8 (1) | 3 (1.2) | 3.3 (1.3) | 3.3 (1.3) |
| LDL (mmol/L) | 1.2 (0.4) | 1.2 (0.4) | 1.2 (0.3) | 1.2 (0.4) | 1.2 (0.3) | 1.2 (0.3) | 1.2 (0.3) | 1.2 (0.3) |
| Triglycerides (mmol/L) | 2.5 (2.2) | 2.5 (2.2) | 2.4 (2.2) | 2.5 (2.3) | 2.5 (2.3) | 2.5 (2) | 2.5 (2.3) | 2.5 (1.9) |

**b) second-line**

|  | 2010 | 2011 | 2012 | 2013 | 2014 | 2015 | 2016 | 2017 |
| --- | --- | --- | --- | --- | --- | --- | --- | --- |
| N | 4387 | 3960 | 3875 | 3792 | 3745 | 4276 | 4258 | 3778 |
| Baseline HbA1c (mmol/mol) | 72 (19) | 74 (20) | 75 (20) | 76 (20) | 76 (19) | 76 (20) | 75 (20) | 75 (20) |
| Age at therapy (years) | 63 (12) | 63 (12) | 63 (12) | 63 (13) | 63 (12) | 63 (12) | 63 (12) | 63 (12) |
| Duration of diabetes (years) | 5 (4) | 5 (4) | 5 (4) | 5 (4) | 5 (4) | 5 (4) | 6 (5) | 6 (5) |
| Sex (% Male) | 60% | 59% | 59% | 60% | 58% | 59% | 58% | 60% |
| Ethnicity (% White/missing) | 94% | 94% | 94% | 93% | 93% | 94% | 93% | 93% |
| BMI (kg/m²) | 32 (7) | 32 (7) | 32 (7) | 32 (7) | 33 (7) | 33 (7) | 33 (7) | 33 (7) |
| Weight (kg) | 93 (22) | 93 (22) | 92 (21) | 92 (22) | 93 (22) | 94 (22) | 94 (22) | 94 (21) |
| eGFR (mL/min/1.73 m²) | 80 (21) | 81 (21) | 80 (21) | 81 (21) | 81 (21) | 82 (21) | 82 (21) | 81 (21) |
| Systolic blood pressure (mmHG) | 135 (16) | 134 (15) | 134 (15) | 134 (15) | 133 (14) | 133 (14) | 134 (14) | 134 (15) |
| Diastolic blood pressure (mmHG) | 78 (10) | 78 (10) | 78 (10) | 78 (10) | 77 (9) | 77 (9) | 78 (9) | 78 (9) |
| HDL (mmol/L) | 2.3 (0.9) | 2.4 (0.9) | 2.4 (1) | 2.4 (1) | 2.4 (1) | 2.6 (1.1) | 2.8 (1.1) | 2.9 (1.2) |
| LDL (mmol/L) | 1.2 (0.4) | 1.2 (0.4) | 1.2 (0.3) | 1.2 (0.3) | 1.2 (0.3) | 1.2 (0.3) | 1.2 (0.3) | 1.2 (0.3) |
| Triglycerides (mmol/L) | 2.3 (1.9) | 2.5 (2.3) | 2.4 (2.2) | 2.4 (1.9) | 2.5 (2.2) | 2.5 (2) | 2.4 (1.8) | 2.5 (2.1) |

**c) third-line**

|  | 2010 | 2011 | 2012 | 2013 | 2014 | 2015 | 2016 | 2017 |
| --- | --- | --- | --- | --- | --- | --- | --- | --- |
| N | 3455 | 3045 | 2871 | 2701 | 2912 | 3413 | 3462 | 3165 |
| Baseline HbA1c (mmol/mol) | 73 (18) | 76 (18) | 76 (18) | 77 (19) | 77 (19) | 78 (18) | 77 (19) | 77 (19) |
| Age at therapy (years) | 63 (11) | 64 (12) | 64 (12) | 64 (12) | 65 (12) | 64 (12) | 64 (12) | 64 (12) |
| Duration of diabetes (years) | 8 (5) | 8 (5) | 8 (5) | 8 (5) | 8 (5) | 8 (5) | 8 (5) | 9 (5) |
| Sex (% Male) | 60% | 59% | 58% | 59% | 59% | 59% | 59% | 58% |
| Ethnicity (% White/missing) | 94% | 94% | 94% | 93% | 94% | 94% | 93% | 92% |
| BMI (kg/m²) | 33 (7) | 33 (7) | 33 (7) | 33 (7) | 33 (7) | 33 (7) | 33 (7) | 33 (7) |
| Weight (kg) | 94 (21) | 94 (22) | 95 (23) | 93 (22) | 93 (22) | 94 (21) | 95 (22) | 93 (21) |
| eGFR (mL/min/1.73 m²) | 79 (21) | 78 (22) | 78 (23) | 77 (23) | 77 (23) | 79 (22) | 79 (22) | 78 (23) |
| Systolic blood pressure (mmHG) | 135 (16) | 135 (16) | 135 (16) | 134 (15) | 133 (15) | 133 (15) | 133 (14) | 134 (15) |
| Diastolic blood pressure (mmHG) | 77 (10) | 77 (10) | 77 (9) | 77 (9) | 76 (9) | 77 (9) | 77 (9) | 77 (9) |
| HDL (mmol/L) | 2.2 (0.8) | 2.2 (0.9) | 2.2 (0.9) | 2.2 (0.9) | 2.2 (0.9) | 2.4 (1) | 2.8 (1.1) | 2.7 (1.2) |
| LDL (mmol/L) | 1.2 (0.4) | 1.2 (0.4) | 1.1 (0.3) | 1.1 (0.3) | 1.2 (0.3) | 1.1 (0.3) | 1.1 (0.3) | 1.1 (0.3) |
| Triglycerides (mmol/L) | 2.2 (1.8) | 2.3 (1.8) | 2.3 (1.7) | 2.3 (1.6) | 2.3 (1.8) | 2.3 (1.6) | 2.5 (2) | 2.4 (1.6) |

**d) fourth-line**

|  | 2010 | 2011 | 2012 | 2013 | 2014 | 2015 | 2016 | 2017 |
| --- | --- | --- | --- | --- | --- | --- | --- | --- |
| N | 2083 | 2031 | 1943 | 1805 | 1980 | 2374 | 2321 | 2143 |
| Baseline HbA1c (mmol/mol) | 76 (19) | 78 (19) | 79 (19) | 81 (20) | 81 (19) | 81 (19) | 81 (19) | 80 (18) |
| Age at therapy (years) | 63 (11) | 64 (10) | 64 (11) | 64 (11) | 64 (11) | 65 (11) | 64 (11) | 64 (11) |
| Duration of diabetes (years) | 10 (5) | 10 (5) | 10 (5) | 10 (6) | 10 (6) | 11 (6) | 11 (6) | 11 (6) |
| Sex (% Male) | 59% | 58% | 58% | 57% | 58% | 59% | 59% | 59% |
| Ethnicity (% White/missing) | 94% | 94% | 95% | 93% | 94% | 95% | 93% | 92% |
| BMI (kg/m²) | 34 (7) | 34 (7) | 33 (7) | 33 (7) | 33 (7) | 33 (7) | 33 (7) | 33 (7) |
| Weight (kg) | 95 (22) | 96 (22) | 96 (22) | 95 (22) | 96 (21) | 95 (22) | 94 (21) | 94 (21) |
| eGFR (mL/min/1.73 m²) | 77 (21) | 77 (21) | 76 (23) | 77 (23) | 76 (24) | 78 (22) | 78 (23) | 77 (23) |
| Systolic blood pressure (mmHG) | 135 (15) | 135 (15) | 135 (16) | 134 (15) | 133 (15) | 133 (15) | 133 (14) | 133 (14) |
| Diastolic blood pressure (mmHG) | 77 (9) | 77 (9) | 76 (10) | 76 (10) | 76 (10) | 76 (9) | 76 (9) | 77 (9) |
| HDL (mmol/L) | 2.2 (0.8) | 2.2 (0.8) | 2.2 (0.9) | 2.1 (0.8) | 2.1 (0.9) | 2.3 (1) | 2.6 (1) | 2.7 (1.1) |
| LDL (mmol/L) | 1.1 (0.4) | 1.2 (0.4) | 1.1 (0.3) | 1.2 (0.3) | 1.1 (0.3) | 1.1 (0.3) | 1.1 (0.3) | 1.1 (0.3) |
| Triglycerides (mmol/L) | 2.3 (2.3) | 2.2 (1.6) | 2.2 (1.8) | 2.2 (1.6) | 2.3 (1.8) | 2.3 (1.5) | 2.4 (2.1) | 2.4 (1.7) |

**Supplementary Table 2: Time trends in hypoglycemia rates for a) first-line b) second-line c) third-line d) fourth-line therapy.**

**a) first-line**

|  | Person-time at risk | Number of events | Rate (per 1000 person-years) | 95% Confidence intervals |
| --- | --- | --- | --- | --- |
| 2010-2011 | 15453 | 48 | 2.91 | 2.08;3.75 |
| 2012-2013 | 15953 | 51 | 2.98 | 2.14;3.81 |
| 2014-2015 | 16631 | 46 | 2.60 | 1.83;3.36 |
| 2016-2017 | 7903 | 25 | 2.93 | 1.77;4.10 |

**b) second-line**

|  | Person-time at risk | Number of events | Rate (per 1000 person-years) | 95% Confidence intervals |
| --- | --- | --- | --- | --- |
| 2010-2011 | 9198 | 74 | 8.22 | 6.32;10.13 |
| 2012-2013 | 8297 | 84 | 10.23 | 8.03;12.42 |
| 2014-2015 | 8396 | 48 | 5.75 | 4.12;7.38 |
| 2016-2017 | 4373 | 25 | 5.70 | 3.46;7.94 |

**c) third-line**

|  | Person-time at risk | Number of events | Rate (per 1000 person-years) | 95% Confidence intervals |
| --- | --- | --- | --- | --- |
| 2010-2011 | 6116 | 63 | 10.62 | 7.94;13.31 |
| 2012-2013 | 5457 | 47 | 8.78 | 6.26;11.30 |
| 2014-2015 | 5974 | 49 | 8.26 | 5.95;10.58 |
| 2016-2017 | 3348 | 30 | 9.01 | 5.79;12.24 |

**d) fourth-line**

|  | Person-time at risk | Number of events | Rate (per 1000 person-years) | 95% Confidence intervals |
| --- | --- | --- | --- | --- |
| 2010-2011 | 2637 | 22 | 8.41 | 4.76;12.07 |
| 2012-2013 | 2405 | 22 | 9.00 | 5.18;12.82 |
| 2014-2015 | 2909 | 19 | 6.57 | 3.60;9.55 |
| 2016-2017 | 1726 | 13 | 7.53 | 3.42;11.65 |

**Supplementary Table 3: Percentage of patients discontinuing a new drug a) within 3 months b) within 6 months c) within 12 months, by calendar year and line of therapy**

**a) within 3 months**

|  | 2010 | 2011 | 2012 | 2013 | 2014 | 2015 | 2016 | 2017 |
| --- | --- | --- | --- | --- | --- | --- | --- | --- |
| First line | 3% | 3% | 3% | 3% | 3% | 3% | 3% | 4% |
| Second line | 9% | 8% | 9% | 8% | 9% | 8% | 9% | 7% |
| Third line | 9% | 9% | 8% | 9% | 10% | 9% | 11% | 12% |
| Fourth line | 9% | 10% | 10% | 10% | 10% | 9% | 11% | 10% |

**b) within 6 months**

|  | 2010 | 2011 | 2012 | 2013 | 2014 | 2015 | 2016 | 2017 |
| --- | --- | --- | --- | --- | --- | --- | --- | --- |
| First line | 4% | 5% | 4% | 4% | 4% | 4% | 5% | NA |
| Second line | 12% | 12% | 13% | 11% | 13% | 13% | 14% | NA |
| Third line | 14% | 14% | 13% | 14% | 14% | 14% | 15% | NA |
| Fourth line | 15% | 15% | 14% | 16% | 14% | 14% | 14% | NA |

**c) within 12 months**

|  | 2010 | 2011 | 2012 | 2013 | 2014 | 2015 | 2016 | 2017 |
| --- | --- | --- | --- | --- | --- | --- | --- | --- |
| First line | 6% | 6% | 6% | 5% | 6% | 6% | 6% | NA |
| Second line | 17% | 17% | 18% | 17% | 18% | 19% | 19% | NA |
| Third line | 20% | 20% | 18% | 20% | 20% | 20% | 20% | NA |
| Fourth line | 21% | 20% | 18% | 22% | 20% | 20% | 19% | NA |

**Supplementary Table 4: Baseline characteristics of included and excluded patients (analysis of HbA1c reduction at 6 months)**

|  | First-line | | Second-line | | Third-line | | Fourth-line | |
| --- | --- | --- | --- | --- | --- | --- | --- | --- |
|  | Included | Excluded | Included | Excluded | Included | Excluded | Included | Excluded |
| N | 26295 | 23920 | 17124 | 14947 | 13428 | 11596 | 8560 | 8120 |
| Baseline HbA1c (mmol/mol) | 71 (21) | 73 (24) | 75 (19) | 75 (21) | 76 (18) | 77 (20) | 79 (18) | 80 (20) |
| Age at therapy (years) | 62 (12) | 62 (13) | 63 (12) | 63 (13) | 64 (11) | 64 (12) | 64 (10) | 64 (11) |
| Duration of diabetes (years) | 2 (3) | 2 (3) | 5 (4) | 5 (5) | 8 (5) | 8 (5) | 10 (5) | 11 (6) |
| Sex (% Male) | 59% | 58% | 60% | 58% | 60% | 58% | 61% | 57% |
| BMI (kg/m²) | 33 (7) | 33 (7) | 33 (7) | 32 (7) | 33 (7) | 33 (7) | 33 (7) | 33 (7) |
| Weight (kg) | 94 (21) | 94 (22) | 93 (21) | 93 (22) | 94 (21) | 93 (23) | 96 (21) | 95 (22) |
| eGFR (mL/min/1.73 m²) | 83 (18) | 82 (19) | 82 (20) | 80 (22) | 79 (22) | 78 (23) | 78 (22) | 76 (23) |
| Systolic blood pressure (mmHG) | 136 (15) | 136 (16) | 134 (14) | 134 (15) | 134 (15) | 134 (15) | 134 (15) | 134 (15) |
| Diastolic blood pressure (mmHG) | 79 (10) | 80 (11) | 78 (9) | 78 (10) | 77 (9) | 77 (10) | 76 (9) | 76 (10) |

**Supplementary Table 5: 6 month weight change and risk of hypoglycemia with and without adjustment for drug as an additional covariate**

**a) Weight change**

| Line of therapy | Annual weight change improvement (kg/year) | Drug adjusted annual weight change improvement (kg/year) |
| --- | --- | --- |
| 1st | -0.09 (-0.13;-0.15), p<0.001 | -0.05 (-0.06;-0.05), p<0.001 |
| 2nd | -0.22 (-0.26;-0.18), p<0.001 | -0.03 (-0.03;-0.02), p<0.001 |
| 3rd | -0.20 (-0.24;-0.15), p<0.001 | -0.05 (-0.05;-0.04), p<0.001 |
| 4th | -0.20 (-0.26;-0.14), p<0.001 | -0.04 (-0.05;-0.03), p<0.001 |

**b) Risk of hypoglycemia**

| Line of therapy | Incidence rate ratio (per year) | Drug adjusted incidence rate ratio (per year) |
| --- | --- | --- |
| 1st | 1.00 (0.93-1.08), p=0.99 | 1.01 (0.94-1.09), p=0.79 |
| 2nd | 0.94 (0.88-1.00), p=0.04 | 0.98 (0.92-1.04), p=0.49 |
| 3rd | 0.96 (0.90-1.02), p=0.21 | 1.02 (0.95-1.09), p=0.62 |
| 4th | 0.95 (0.85-1.06), p=0.35 | 0.99 (0.88-1.12), p=0.90 |

**Supplementary Figure 1: Time trends (2010-2017) in new drug prescriptions across lines 1-4of therapy (n=123,990).**The prescriptions for each drug class each year are given as a percentage of total new drug prescriptions for that year.


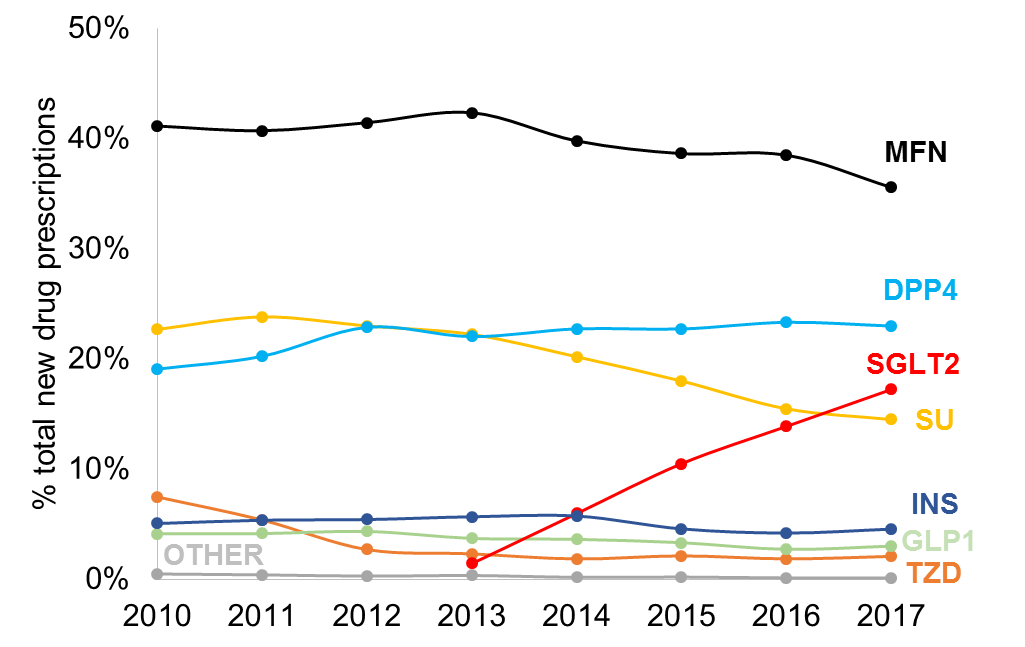


**Supplementary Figure 2: Time trends (2014-2017) in new within class drug prescriptions across all lines of therapy for a) DPP4 inhibitors b) GLP-1 agonists c) SGLT2 inhibitors d) sulfonylureas.** The prescriptions for each drug subtype each year are given as a percentage of total new prescriptions of the drug class for that year.

**a) DPP4 inhibitors (n=29,835)**

**
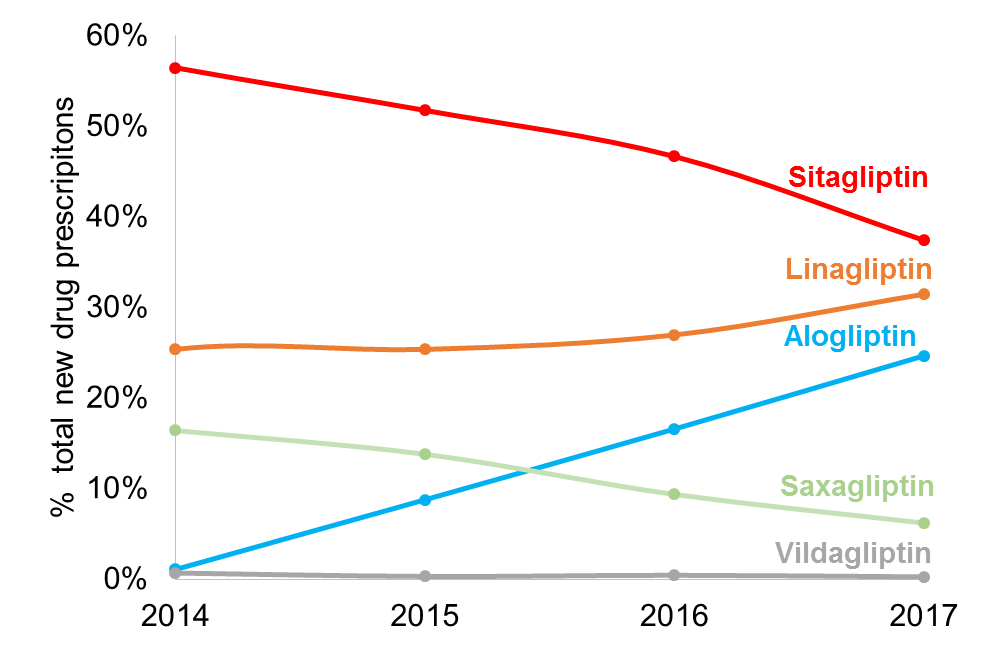
**

**b) GLP-1 receptor agonists (n=6,989)**

**
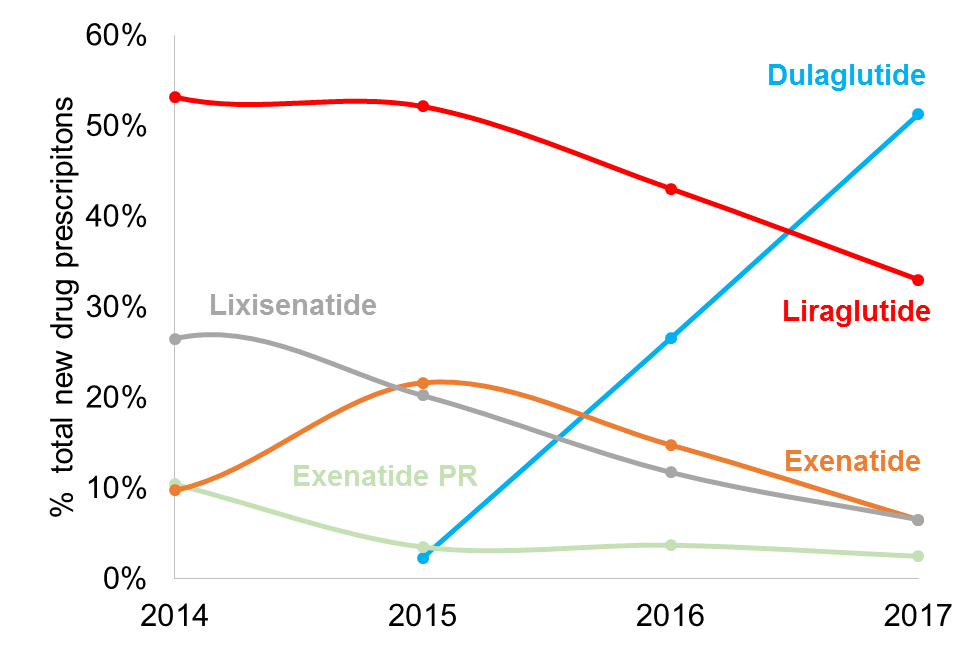
**

**c) SGLT2 inhibitors (n=11,255)**

**
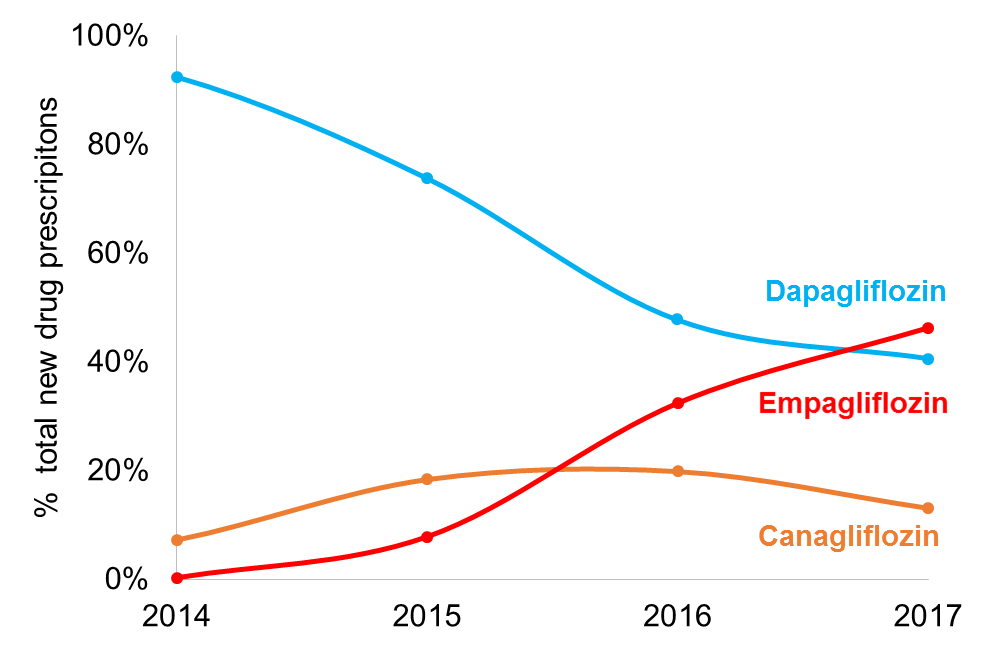
**

**d) Sulfonylureas (n=24,506)**

**
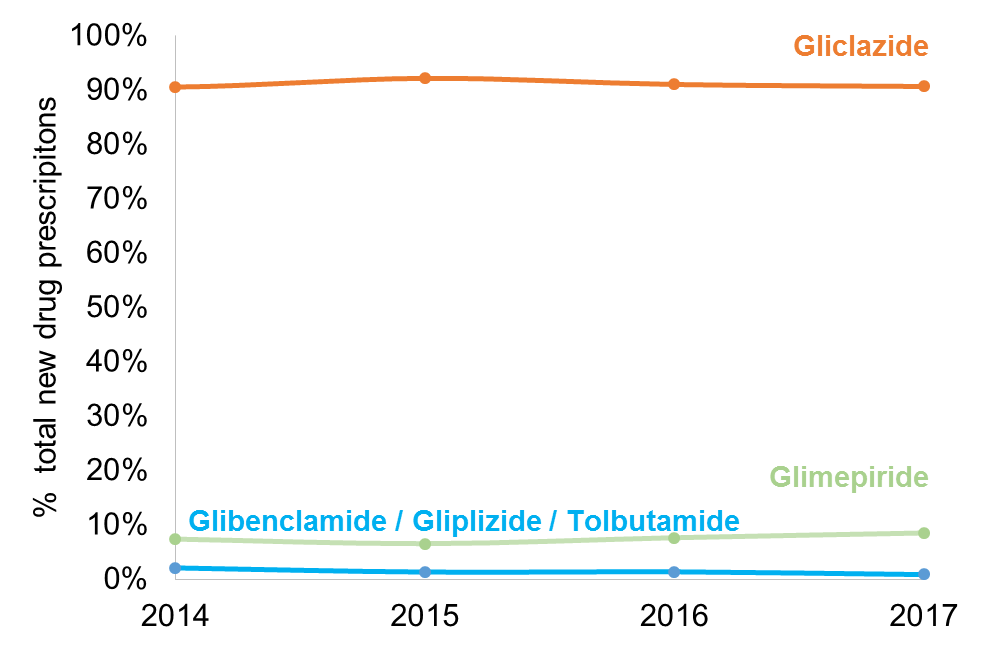
**

**Supplementary Figure 3a: Mean change in systolic blood pressure (SBP) at 6 months, 2010-2017 for a) first-line b) second-line c) third-line d) fourth-line.** Error bars are 95% confidence intervals. Data are standardised to the mean baseline systolic blood pressure, baseline HbA1c, age at diagnosis and duration of diabetes over the entire study period, specific to each drug line.


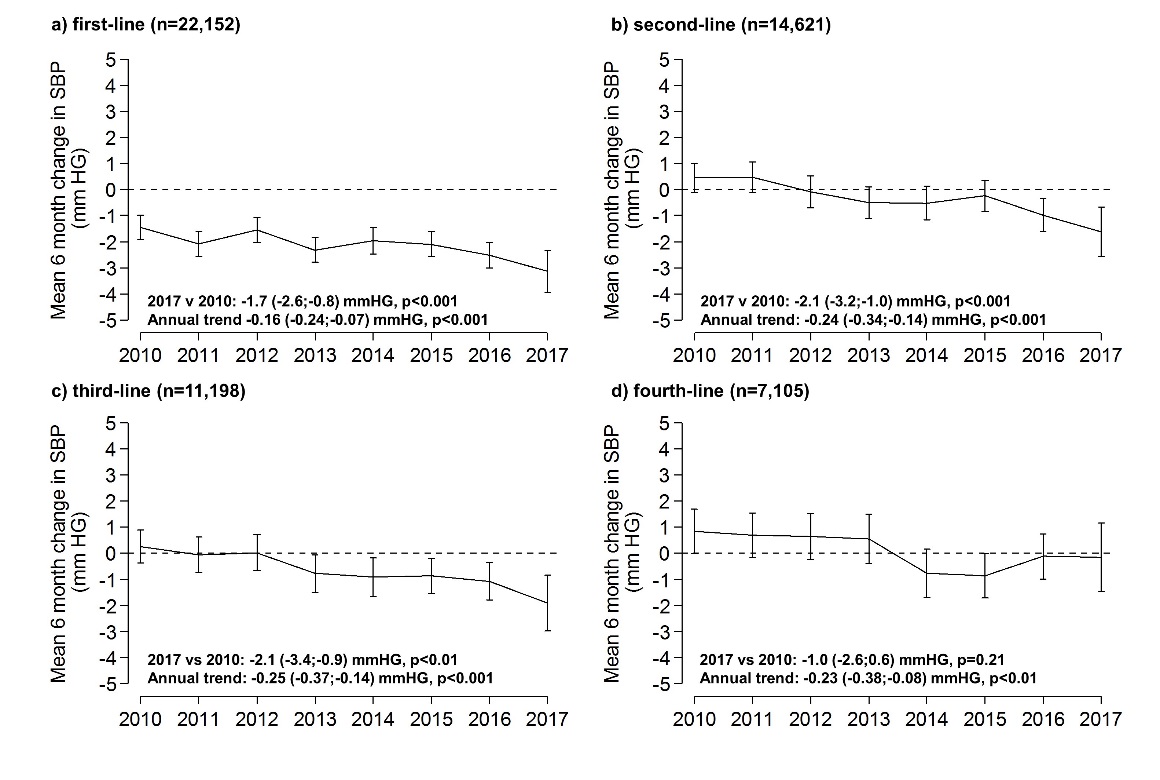


**Supplementary Figure 3b: Mean change in diastolic blood pressure (DBP) at 6 months, 2010-2016 for a) first-line b) second-line c) third-line d) fourth-line therapy.** Error bars are 95% confidence intervals. Data are standardised to the mean baseline diastolic blood pressure, baseline HbA1c, age at diagnosis and duration of diabetes over the entire study period, specific to each drug line.


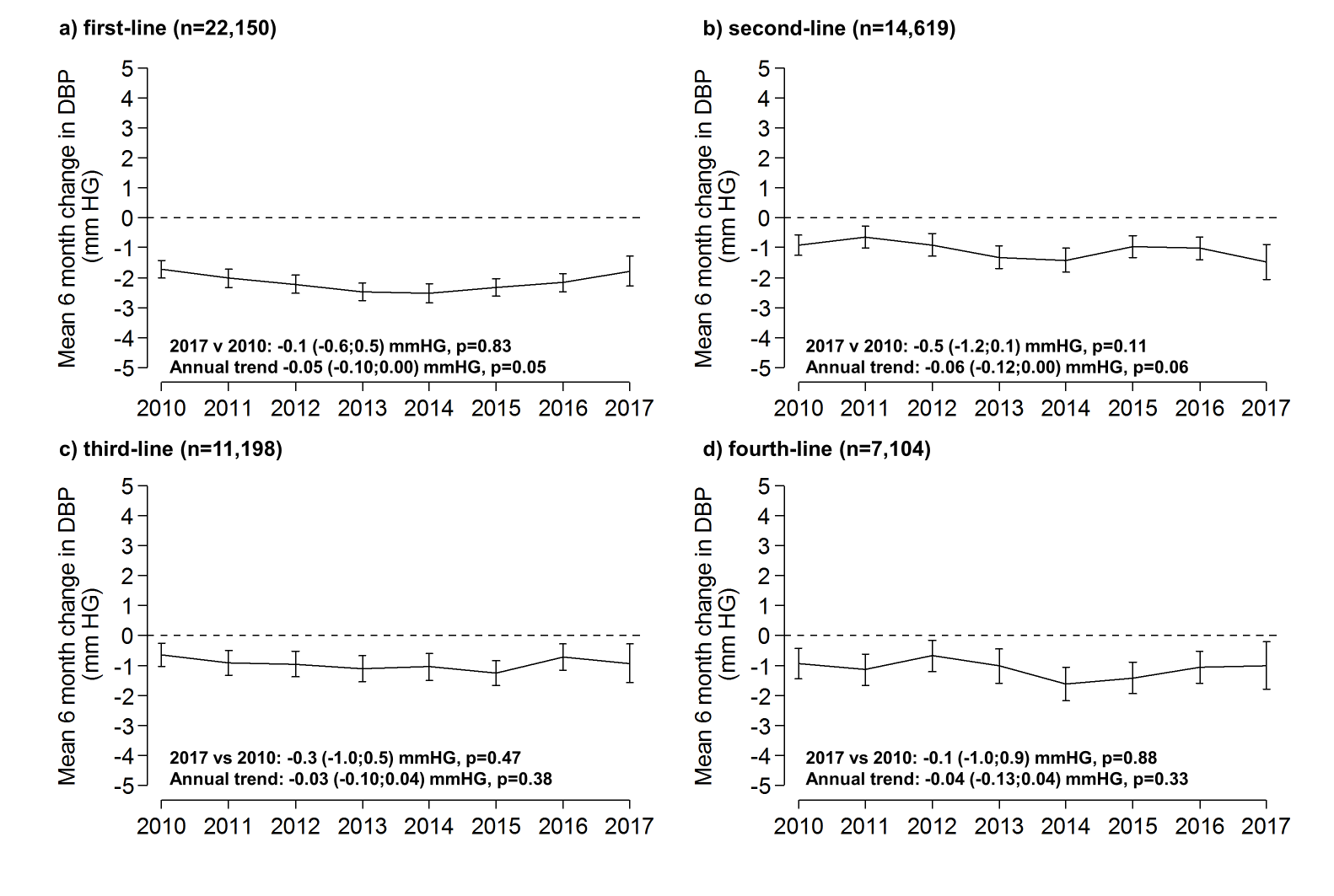


**Supplementary Figure 4: Mean change in HbA1c at 12 months, 2010-2016 for a) first-line b) second-line c) third-line d) fourth-line.** Error bars are 95% confidence intervals. Data are standardised to the mean baseline HbA1c, age at diagnosis and duration of diabetes over the entire study period, specific to each drug line.


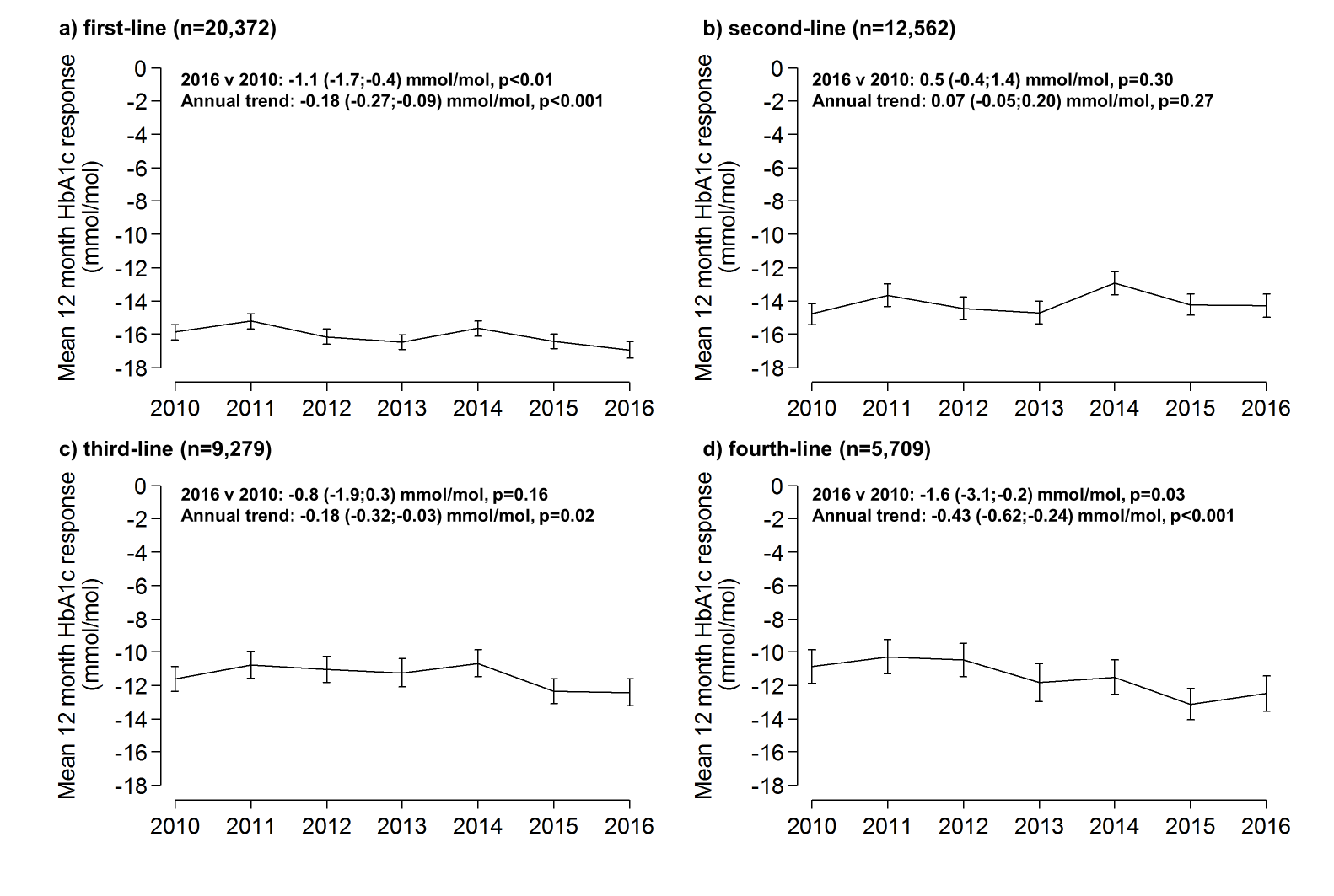


**Supplementary Figure 5: Mean change in weight at 12 months, 2010-2016 for a) first-line b) second-line c) third-line d) fourth-line.** Error bars are 95% confidence intervals. Data are standardised to the mean baseline weight, baseline HbA1c, age at diagnosis and duration of diabetes over the entire study period, specific to each drug line.


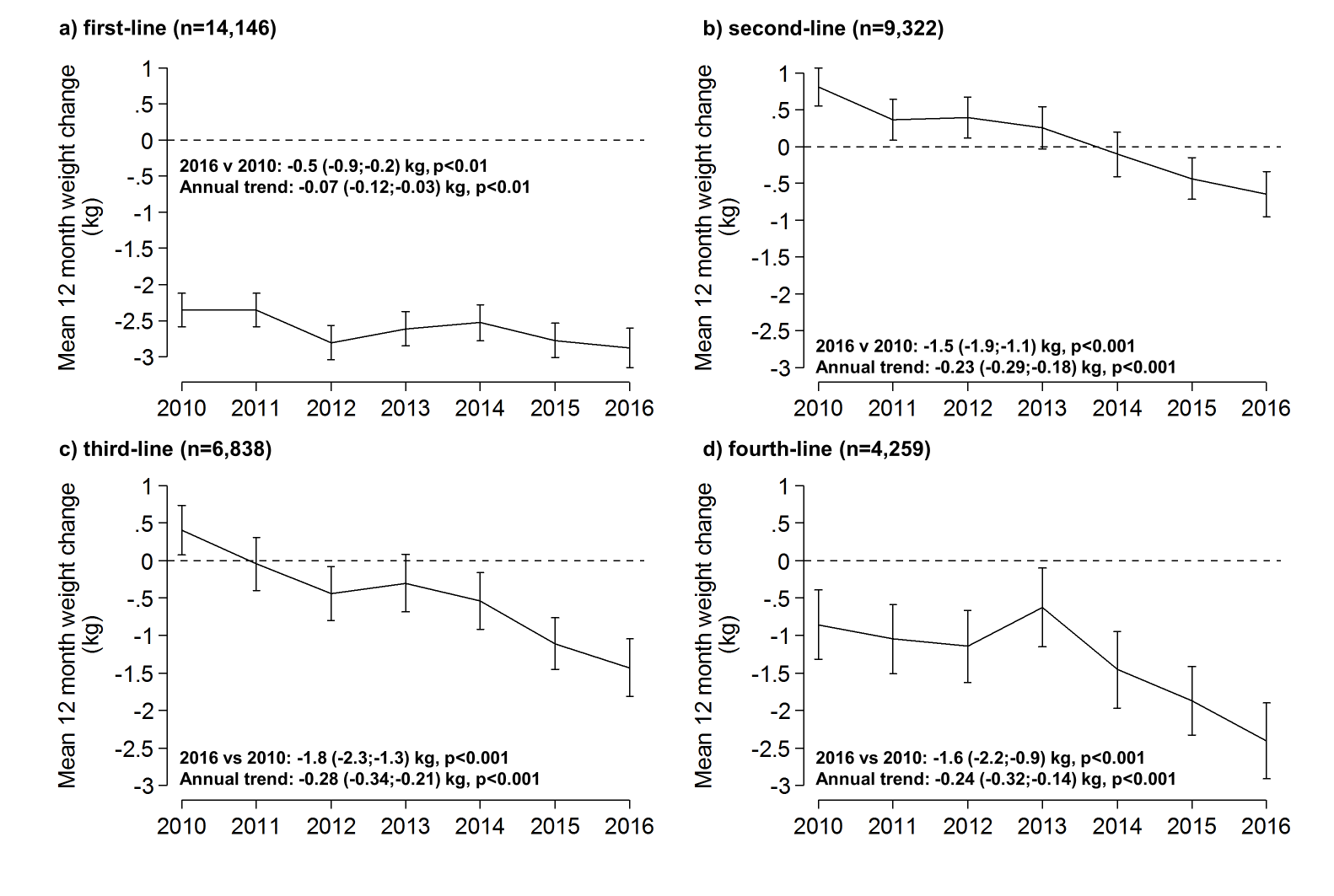


**Supplementary Figure 6a: Mean change in systolic blood pressure (SBP) at 12 months, 2010-2016 for a) first-line b) second-line c) third-line d) fourth-line.** Error bars are 95% confidence intervals. Data are standardised to the mean baseline systolic blood pressure, baseline HbA1c, age at diagnosis and duration of diabetes over the entire study period, specific to each drug line.


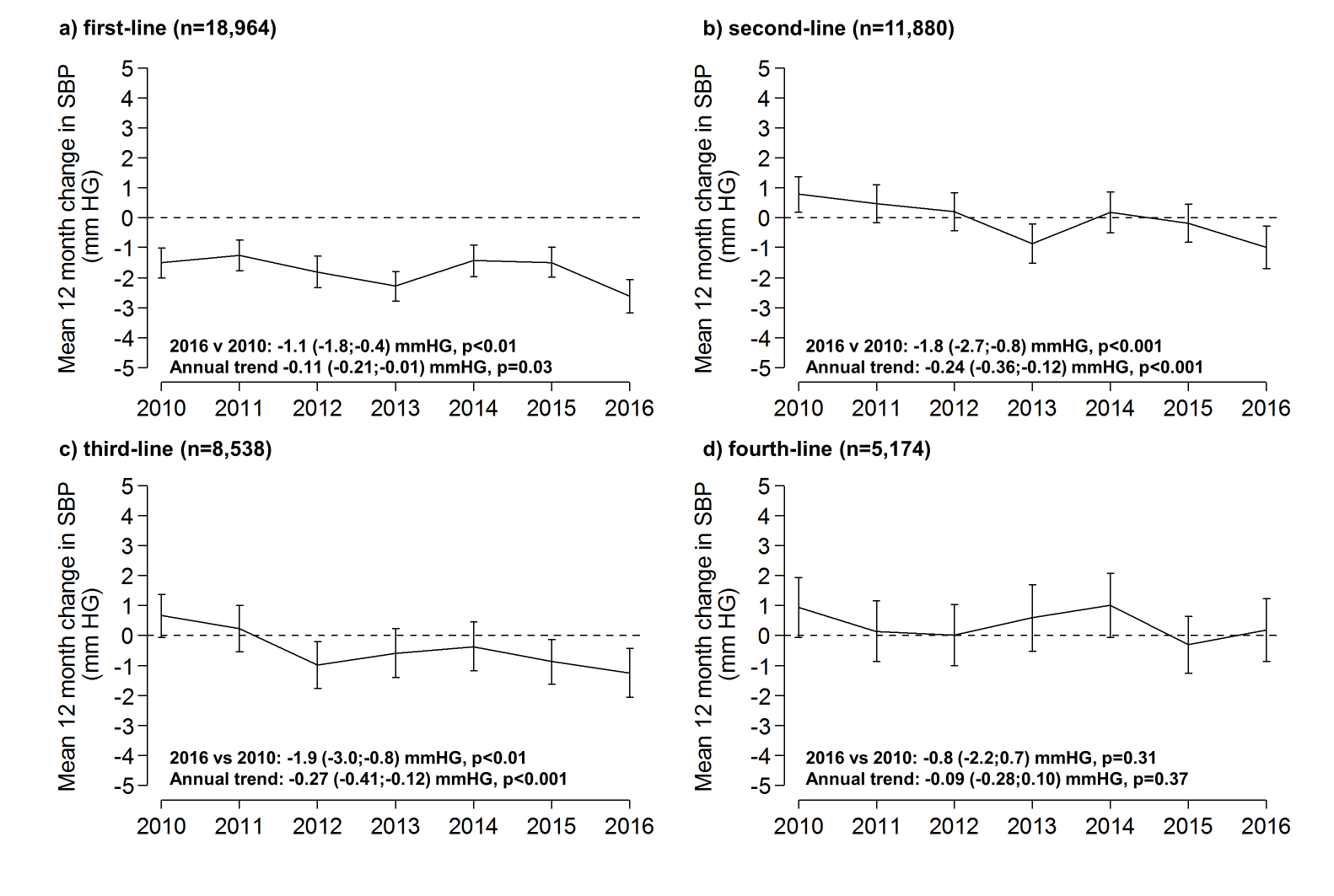


**Supplementary Figure 6b: Mean change in diastolic blood pressure (DBP) at 12 months, 2010-2016 for a) first-line b) second-line c) third-line d) fourth-line therapy.** Error bars are 95% confidence intervals. Data are standardised to the mean baseline diastolic blood pressure, baseline HbA1c, age at diagnosis and duration of diabetes over the entire study period, specific to each drug line.


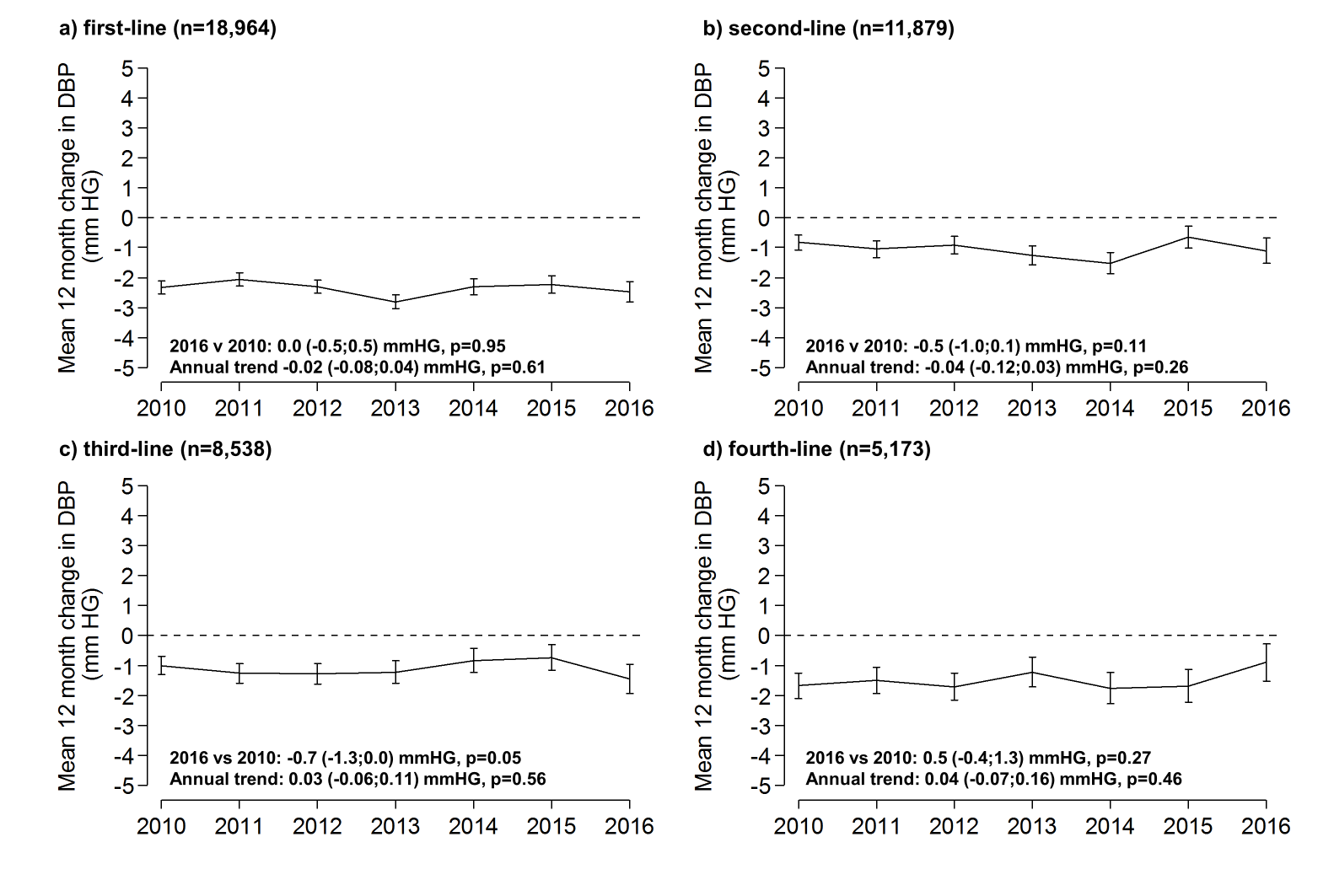


**Supplementary Figure 7: Second-line prescribing trends and patient outcomes in the subset of patients adding a second-line drug to continued first-line metformin therapy (73% of patients included in the primary analysis)**

**a) Time trends in new drug prescriptions.** The prescriptions for each drug class each year are given as a percentage of total new drug prescriptions for that year.

**
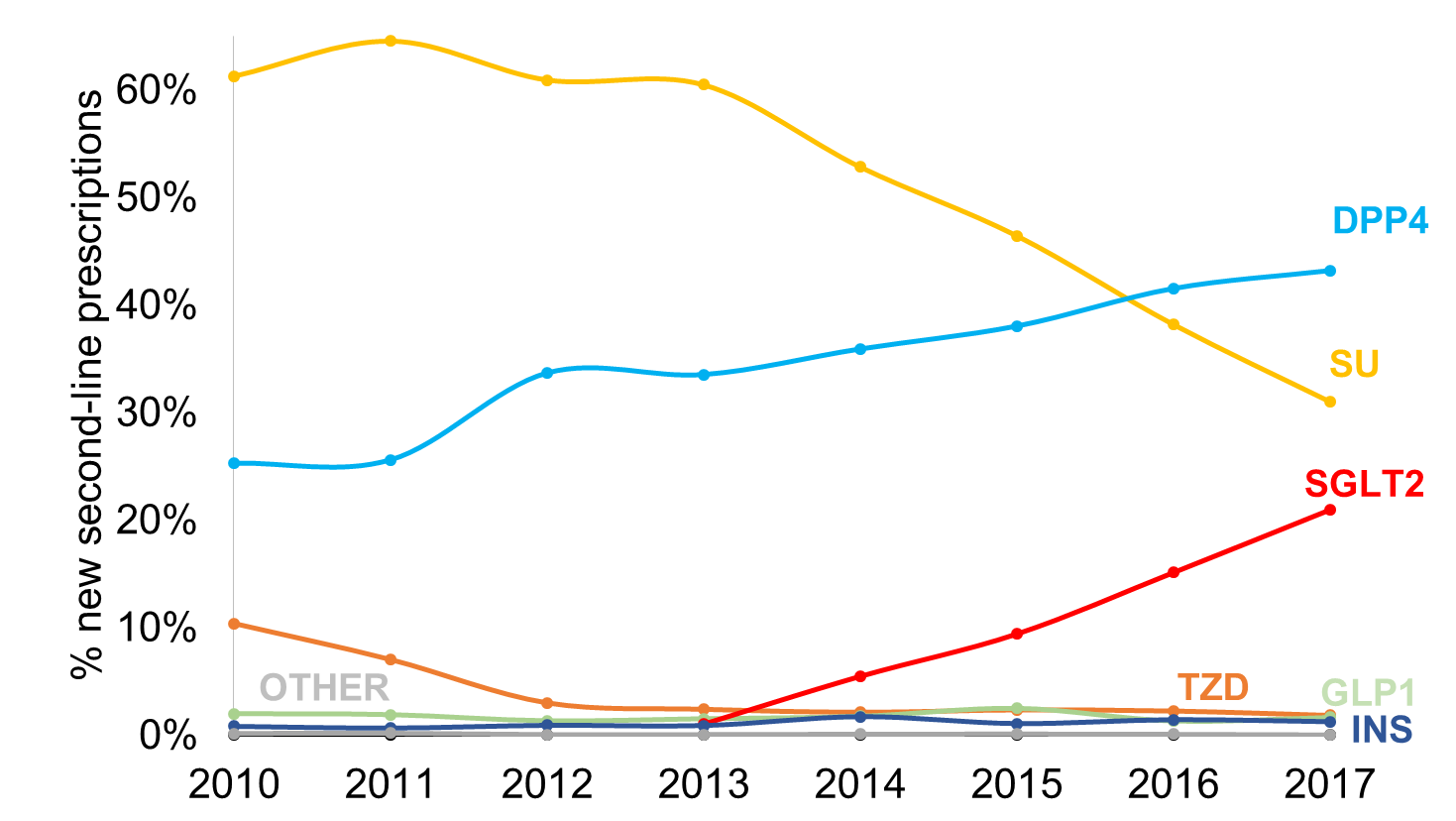
**

**b) Mean change in HbA1c at 6 months, 2010-2017.** Error bars are 95% confidence intervals. Data are standardised to the average baseline HbA1c, age at diagnosis and duration of diabetes in 2017.

**c) Mean change in weight at 6 months, 2010-2017 for a) first-line b) second-line c) third-line d) fourth-line.** Error bars are 95% confidence intervals. Data are standardised to the average baseline HbA1c, age at diagnosis and duration of diabetes in 2017.

**d) Hypoglycemia rates per 1,000 years by 2 year period.** Rates represent the occurrence of hypoglycemia over the first two years after starting second-line therapy.


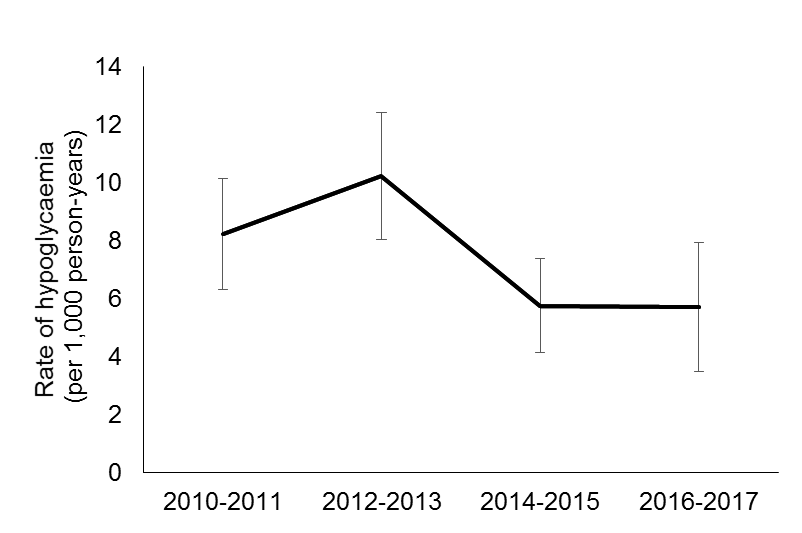

Supplement: Supplementary file 1 — Appendix [file DOM-21-1576-s001.docx]
